# The prognostic value of CXC chemokine receptor 2 (CXCR2) in cancers: a meta-analysis

**DOI:** 10.18632/oncotarget.23492

**Published:** 2017-12-11

**Authors:** Bingbing Qiao, Wenqin Luo, Yanna Liu, Jing Wang, Chuan Liu, Zhao Liu, Sizhe Chen, Jingjing Gu, Xiaolong Qi, Tongwei Wu

**Affiliations:** ^1^ Department of Hepatobiliary Surgery, The First Affiliated Hospital of Zhengzhou University, Zhengzhou, China; ^2^ Department of General Surgery, Nanfang Hospital, Southern Medical University, Guangzhou, China; ^3^ Department of Hepatobiliary Disease, The Affiliated (T.C.M) Hospital of Southwest Medical University, Luzhou, China

**Keywords:** CXCR2, cancer, overall survival, recurrence-free survival, prognostic

## Abstract

**Background & Aims:**

Quite a few studies had investigated the correlation between CXC chemokine receptor 2 (CXCR2) and cancer. This meta-analysis was aimed to comprehensively summarize the previous studies and to explore the prognostic value of CXCR2 in patients with cancer.

**Materials and Methods:**

An adequate literature search in EMBASE and PubMed was conducted. Articles in English which have reported CXCR2 expression in patients and enough data to calculate hazard ratio (HR) were included. Effect estimates were analyzed with Review Manager 5.2. The endpoint was overall survival (OS) and recurrence-free survival (RFS).

**Result:**

Twelve studies from 10 publications with a total of 2,461 patients were identified. It was shown that high level of CXCR2 was significantly associated with poorer overall survival (OS) (HR = 1.69, 95% CI = 1.46–1.96, *p* < 0.0001, I^2^ = 45%) and RFS (HR = 1.50, 95% CI = 1.25–1.80, *p* < 0.0001, I^2^ = 6%). The analyses of different analysis models (univariate or multivariate models), sample size (< 300 or ≥ 300) and ethnicity (Asian and Caucasian) have indicated the negative impact of CXCR2 over-expression on survival of patients with cancer. Stratified by cancer type, high-expression of CXCR2 was associated with unfavorable OS in laryngeal squamous cell carcinoma, lung cancer, pancreatic ductal carcinoma, clear-cell renal cell carcinoma and hepatocellular carcinoma; however, there was significant difference between high- and low-expression of CXCR2 in digestive tract cancer (esophageal adenocarcinoma and squamous cell carcinoma procession, resected esophageal carcinoma, esophageal cancer and gastric cancer).

**Conclusions:**

CXCR2 is an unfavorable predictor in terms of OS and RFS in patients with cancer except for digestive tract cancer and is related with poorer prognostic.

## INTRODUCTION

Cancer is the leading cause of death in both developing and developed countries. Meanwhile, both incidence and mortality of cancer grow rapidly for increasing population, senility, and unhealthy adoption of lifestyle behaviors [1]. It is identified that surgery, radiotherapy and chemotherapy are utilized as standard treatments in cancer cases. Nevertheless, most patients still have poor prognostic.

Chemokines, a large family of small and structurally related protein molecules with well-recognized roles in directional recruitment and migration of cells [2], have been implicated in migration of leukocytes, embryogenesis, angiogenesis, hematopoiesis, atherosclerosis, tumor growth and metastasis. There are four subgroups of chemokines: CXC, CC, CX3C, and C chemokine ligands. CXC chemokines are further subdivided into ELR + CXC chemokines and ELR - CXC chemokines.

CXC chemokine receptor 2 (CXCR2), an ELR + CXC chemokine receptor, was responsible for the angiogenic activity and endothelial cell chemotaxis [3]. Besides, CXCR2, also a receptor for IL-8, can regulate neutrophil migration to sites of inflammation [4]. Due to its role in recruiting myeloid cells to the tumor microenvironment and to the metastatic niche, CXCR2 may be engaged in tumorigenesis and the metastatic process [5]. In addition, previous studies have demonstrated that CXCR2 plays a critical role in cancers, such as lung cancer [6, 7], laryngeal squamous cell carcinoma [8], astrocytic tumors [9], pancreatic ductal carcinoma [10], clear-cell renal cell carcinoma [11] and hepatocellular carcinoma [12]. An *et al.* 2015 [11], had indicated that CXCR2 was correlated with poor prognosis and could be used as a novel prognostic factor in patients with non-metastatic clear-cell renal cell carcinoma (ccRCC). Moreover, Hertzer *et al.* 2013, had pointed out that CXCR2 could be considered as a target for pancreatic cancer treatment [13]. Varied in tumor types, although previous studies have drawn such a general consensus, no meta-analysis was conducted to analyze the prognosis value in cancer comprehensively. Therefore, this study, aimed to summarize prominent studies and determine the clinical efficacy of CXCR2 in predicting the prognosis of cancer patients, was carried out.

## MATERIALS AND METHODS

### Literature search strategy

PubMed and EMBASE database were searched up to October 25th, 2016. The search strategy was “CXCR2 or interleukin-8 receptor 2” in combination with “cancer”. All eligible studies were retrieved, and their reference lists were checked for additional relevant publications.

### Inclusion criteria

Studies researching comparisons of CXCR2 expression in patients with different types of cancer were eligible for inclusion. Studies that met all the following criteria were included: (i) English articles; (ii) reporting CXCR2 expression in cancer patients; (iii) enough data on the expression of CXCR2 or overall survival (OS) or recurrence-free survival (RFS) to calculate hazard ratio (HR).

### Exclusion criteria

The exclusion criteria were as follows: (i) not focusing on the expression of CXCR2 in cancer patients; (ii) ongoing studies; (iii) review articles.

### Data extraction

As for each study, the following information was extracted: year of publication, country, ethnicity, number of patients, the percentage of male and tumor type. Data extraction and information on study design, outcomes were performed by two independent reviewers (Zhao Y and Luo W) and disagreements were resolved by discussion and consensus with a third reviewer (Qi X).

### Statistical analysis

The specify guidelines we followed for this meta-analysis was PRISMA. Effect estimates were analyzed with Review Manager 5.2. Dichotomous data were compared using HR. The HRs with their 95% confidence interval (CI) s were directly obtained from the article or calculated by using previously published methods [14]. Forest plots were generated for graphical presentations, and heterogeneity among different studies was appraised by I^2^ estimates. Fixed-effects model was used to aggregate data if there were no statistical heterogeneity (*p* > 0.1 or I^2^ < 50%) otherwise randomized-effects model was conducted. Publication reporting bias was visually evaluated using the funnel plot. The difference was significant when *p* < 0.05.

## RESULTS

### Literature search

**Figure 1:**
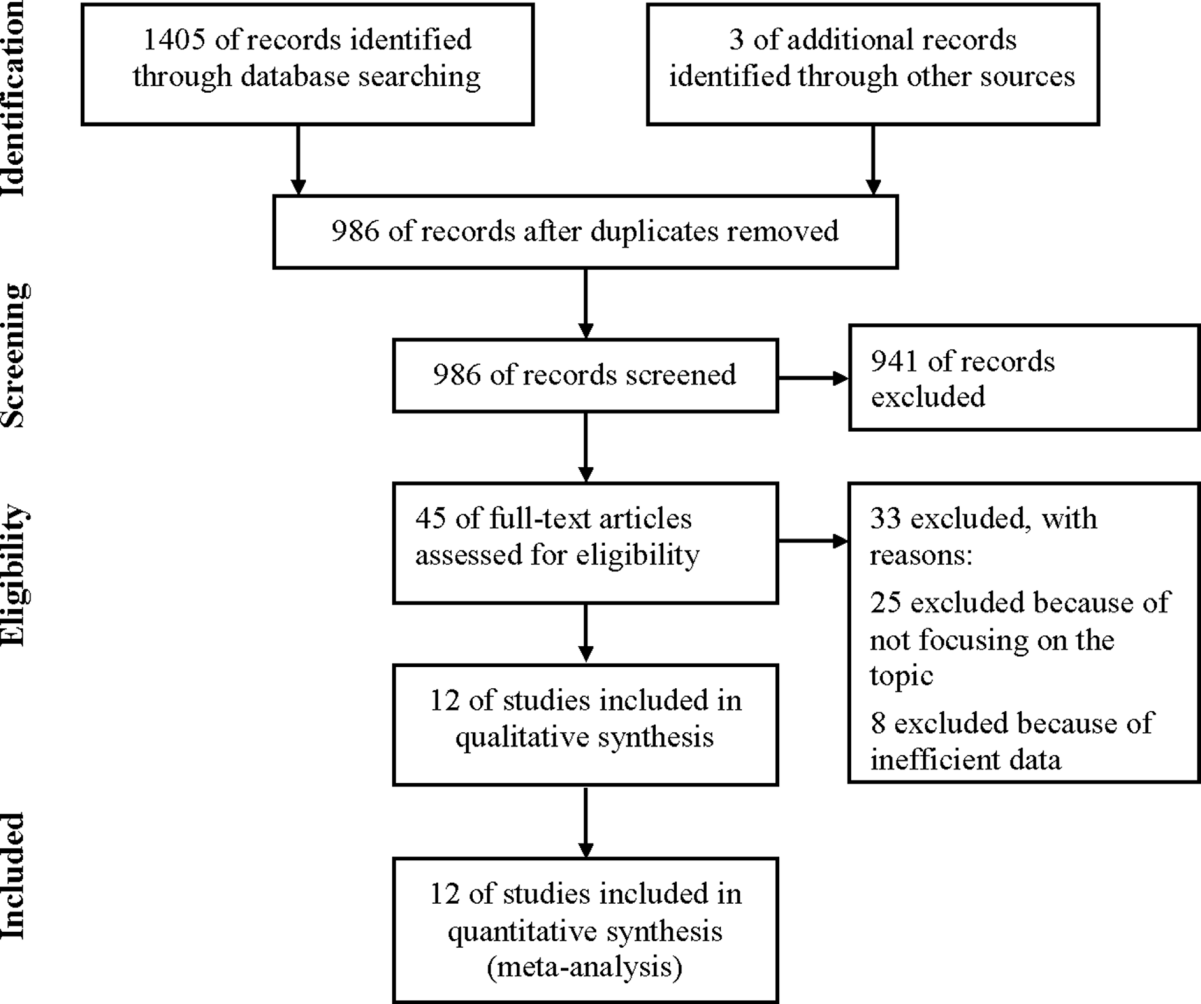
Flow diagram of study selection process

As shown in Figure 1, 1,408 initial articles that included our search terms were obtained. Of those, 986 of records were screened after duplicates removed. The rest 45 articles were retrieved for full-text review, from which 33 were excluded: 25 not focusing on the expression of CXCR2 in cancer patients and 8 without efficient data on the expression of CXCR2 or overall survival (OS) or recurrence-free survival (RFS) to calculate HR. Finally, 12 studies met the inclusion criteria and were included in qualitative synthesis.

### Characteristics of included studies

The detailed characteristics of included studies were listed in Table 1. The studies were conducted in 5 countries (9 Asian cohorts and 3 Caucasian cohorts), and they were published between 2012 and 2016. Studies concerning 5 digestive tract cancer (1 esophageal adenocarcinoma and squamous cell carcinoma procession cohort [15], 1 resected esophageal carcinoma cohort [16], 1 esophageal cancer cohort and 2 gastric cancer cohorts [17, 18]) occupied the largest proportion of cancer type among all primary literatures, followed by lung cancer (*n =* 2) [6, 7], then laryngeal squamous cell carcinoma (*n =* 1) [8], astrocytic tumors (*n =* 1) [9], pancreatic ductal carcinoma (*n =* 1) [10], clear-cell renal cell carcinoma (*n =* 1) [11] and hepatocellular carcinoma (*n =* 1) [12]. All studies reported the OS while only four studies reported the RFS. Almost all the included studies used multivariate models except for two studies conducted by Sui *et al.* [16] and Nishi *et al.* [19], respectively, with univariate models.

**Table 1:** Characteristic of studies

Study Name	Year	Country	Ethnicity	Patients (n)	Male (%)	Outcome	Tumor	Analysis
Han et al.	2012	China	Asian	109	98.16%	OS	laryngeal squamous cell carcinoma	M
Korkolopoulou et al.	2012	Greece	Caucasian	120	76.70%	OS	Astrocytic Tumors	M
Saintigny et al.	2013	Texas	Caucasian	262	48.90%	OS	lung adenocarcinoma	M
Gold et al.	2014	America	Caucasian	370	50.30%	OS, RFS	Non–Small Cell Lung	M
Liang et al.	2014	China	Asian	159	50.31%	OS	esophageal adenocarcinoma and squamous cell carcinoma procession	M
Sui et al.	2014	China	Asian	95	82.10%	OS	resected esophageal carcinoma	U
Wang et al.	2014	China	Asian	161	53.42%	OS	pancreatic ductal carcinoma	M
An et al.	2015	China	Asian	375	71.20%	OS, RFS	clear-cell renal cell carcinoma	M
Li et al.	2015	China	Asian	259	87.25%	OS, RFS	hepatocellular carcinoma	M
Nishi et al.	2015	Japan	Asian	82	89.00%	OS, RFS	esophageal cancer	U
Wang et al.	2015	China	Asian	357	70.00%	OS	gastric cancer	M
Yang et al.	2016	China	Asian	112	66.96%	OS	gastric adenocarcinoma	M

### Study quality

As presented in Table 2, the quality of each study included in this meta-analysis was graded with Newcastle-Ottawa scale, a score between 0–9 for each study (studies with score of 6–9 manifesting good quality studies). All studies included in this study had the score over 7, which indicated good quality.

**Table 2:** Quality indicators from the Newcastle-Ottawa scale

Study	Selection				Comparable		Outcome assessment			
	1	2	3	4	1	2	1	2	3	SCORE
An et al. 2015	*	*	*	*	*	*	*	*	*	9
Gold et al. 2014	*	*	*	*	*	*	*	*		8
Han et al. 2012	*	*	*	*	*	*	*	*		8
Korkolopoulou et al. 2012	*	*	*		*		*	*	*	8
Li et al. 2015	*	*	*	*	*	*	*	*	*	9
Liang et al. 2014	*	*	*		*	*	*	*		7
Nishi et al. 2015	*	*	*		*	*	*	*	*	8
Saintigny et al. 2013	*	*	*		*	*	*	*	*	8
Sui et al. 2014	*	*	*		*	*	*		*	7
Wang et al. 2014	*	*		*	*	*	*	*	*	8
Wang et al. 2015	*	*		*	*	*	*	*	*	8
Yang et al. 2016	*	*	*		*	*	*	*		7

### Meta-analyses of RFS

**Figure 2:**
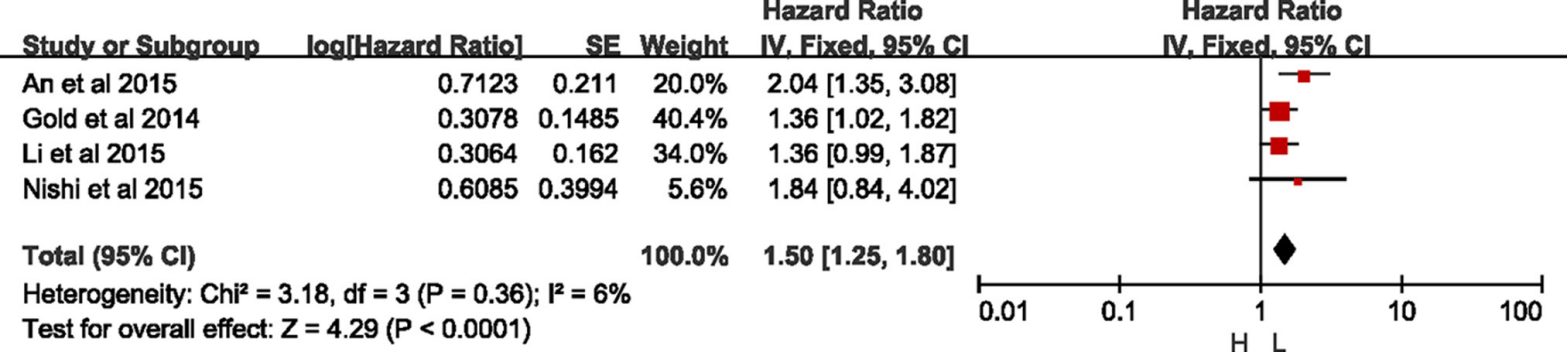
Meta-analysis of recurrence-free survival

Four eligible studies reported the RFS [7, 11, 12, 19, 20]. There was no significant heterogeneity between these studies when pooling the HR, so HR was pooled in the fixed-effects model. As Figure 2 shown, the results showed that higher expression of CXCR2 was correlated with shorten RFS (HR = 1.50, 95% CI = 1.25–1.80, *p* < 0.0001; I^2^ = 6%, *p* = 0.36). Besides, there was no bias among all included studies from the funnel plot. (Supplementary Figure 1).

### Meta-analyses of OS

**Figure 3:**
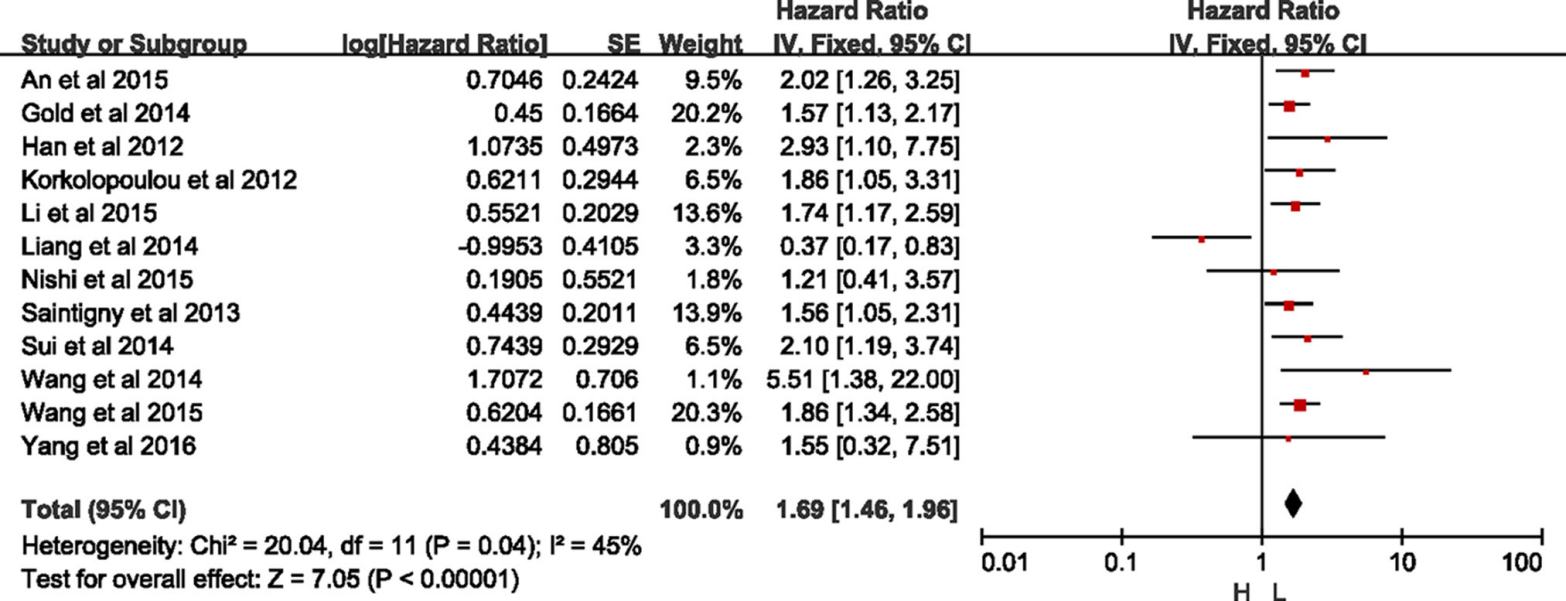
Meta-analysis of overall survival

As Figure 3 presented, twelve studies offered original data of OS. The synthesis showed that over-expression of CXCR2 was significantly related to a poorer OS (HR = 1.69, 95% CI = 1.46–1.96, *p* < 0.0001). Because heterogeneity was detected (I^2^ = 45%, *p* = 0.04), a randomized-effects model was used to determine the pooled HR and 95% CI. The funnel plot presented no bias among all included studies (Supplementary Figure 2).

As Table 3 shown, high-expression of CXCR2 was significantly related to a poorer OS for patients with laryngeal squamous cell carcinoma (HR = 2.93, 95% CI = 1.10–7.75, *p* = 0.03) in the subgroup analysis by tumor type, astrocytic tumors (HR = 1.86, 95% CI = 1.05–3.31, *p* = 0.03), lung cancer (HR = 1.56, 95% CI = 1.22–2.01, *p* = 0.0005; I^2^ = 0%, *p* = 0.98), pancreatic ductal carcinoma (HR = 5.51, 95% CI = 1.38–22.0, *p* = 0.02), clear-cell renal cell carcinoma (HR = 2.20, 95% CI = 1.26–3.25, *p* = 0.004) and hepatocellular carcinoma (HR = 1.74, 95% CI = 1.17–2.59, *p* = 0.007), respectively. However, high-expression of CXCR2 in digestive tract cancer (HR = 1.26, 95% CI = 0.68–2.35, *p* = 0.46; I^2^ = 73%, *p* = 0.005) has no effect on OS statistically.

**Table 3:** Subgroup analysis of OS

Survival analysis	Included cohorts	HR 95% CI	*p*	I^2^	*p* value for heterogeneity
**Analysis model**					
Multivariate	10	1.66 [1.30, 2.14]	< 0.00001	53%	0.02
Univariate	2	1.86 [1.12, 3.09]	0.02	0%	0.38
**Ethnicity**					
Caucasian	3	1.61 [1.28, 2.02]	< 0.00001	0%	0.86
Asian	9	1.69 [1.20, 2.39]	0.003	59%	0.01
**Sample Size**					
< 300	9	1.61 [1.13, 2.32]	0.009	58%	0.02
≥ 300	3	1.76 [1.43, 2.17]	< 0.00001	0%	0.63
**Tumor**					
LSCC	1	2.93 [1.10, 7.75]	0.03	NA	NA
Astrocytic Tumors	1	1.86 [1.05, 3.31]	0.03	NA	NA
Lung cancer	2	1.56 [1.22, 2.01]	0.0005	0	0.98
EACC/ESCC/ ECC/GC	5	1.26 [0.68, 2.35]	0.46	73%	0.005
PDAC	1	5.51 [1.38, 22.0]	0.02	NA	NA
RCC	1	2.20 [1.26, 3.25]	0.004	NA	NA
HCC	1	1.74 [1.17, 2.59]	0.007	NA	NA

OS, overall survival; LSCC, laryngeal squamous cell carcinoma; EACC/ESCC, esophageal adenocarcinoma and squamous cell carcinoma procession; ECC, resected esophageal carcinoma; PDAC, pancreatic ductal carcinoma; RCC, clear-cell renal cell carcinoma; HCC, hepatocellular carcinoma; ESCC, esophageal cancer; GC, gastric cancer; NA, not applicable.

**Figure 4:**
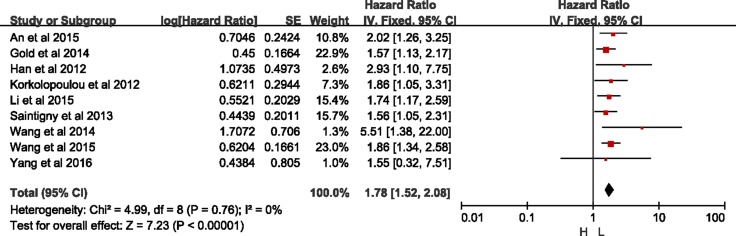
Meta-analysis of group with multivariate model

Among the subgroup, two studies were demonstrated in univariate models (HR = 1.86, 95% CI = 1.12–3.09, *p* = 0.02; I^2^ = 0%, *p* = 0.38) without heterogeneity, and ten in multivariate models (HR = 1.66, 95% CI = 1.30–2.14, *p* < 0.01; I^2^ = 53%, *p* = 0.02), with significant difference but with heterogeneity. With sensitivity analysis, the study by Liang *et al.* [15] was excluded with regard of OS analysis with multivariate models (Supplementary Figure 3). As shown in Figure 4, significant difference was detected between high- and low-expression of CXCR2 without heterogeneity in group with multivariate models (HR = 1.78, 95% CI = 1.52–2.08, *p* < 0.01; I^2^ = 0%, *p* = 0.76).

**Figure 5:**
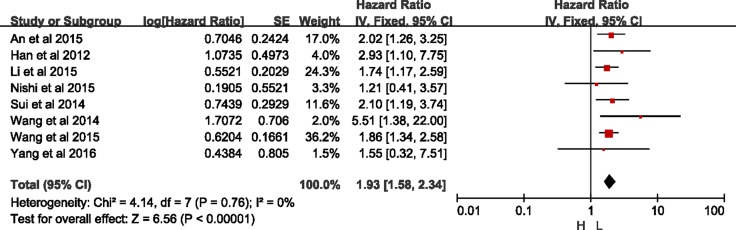
Meta-analysis of Asian group

Besides, subgroup analyses by ethnicity revealed that CXCR2 was an unfavorable predictor of OS both in Asian populations along with a significant heterogeneity (HR = 1.69, 95% CI = 1.20–2.39, *p* = 0.003; I^2^ = 59%, *p* = 0.01), and in Caucasian populations (HR = 1.61, 95% CI = 1.28–2.02, *p* < 0.00001; I^2^ = 0%, *p* = 0.86) (Table 2). With sensitivity analysis, the study by Liang *et al.* [15] was excluded with regard of OS analysis in Asian group (Supplementary Figure 4). As shown in Figure 5, there was significant difference between high- and low-expression of CXCR2 without heterogeneity in Asian group (HR = 1.93, 95% CI = 1.58–2.34, *p* < 0.01; I^2^ = 0%, *p* = 0.76).

**Figure 6:**
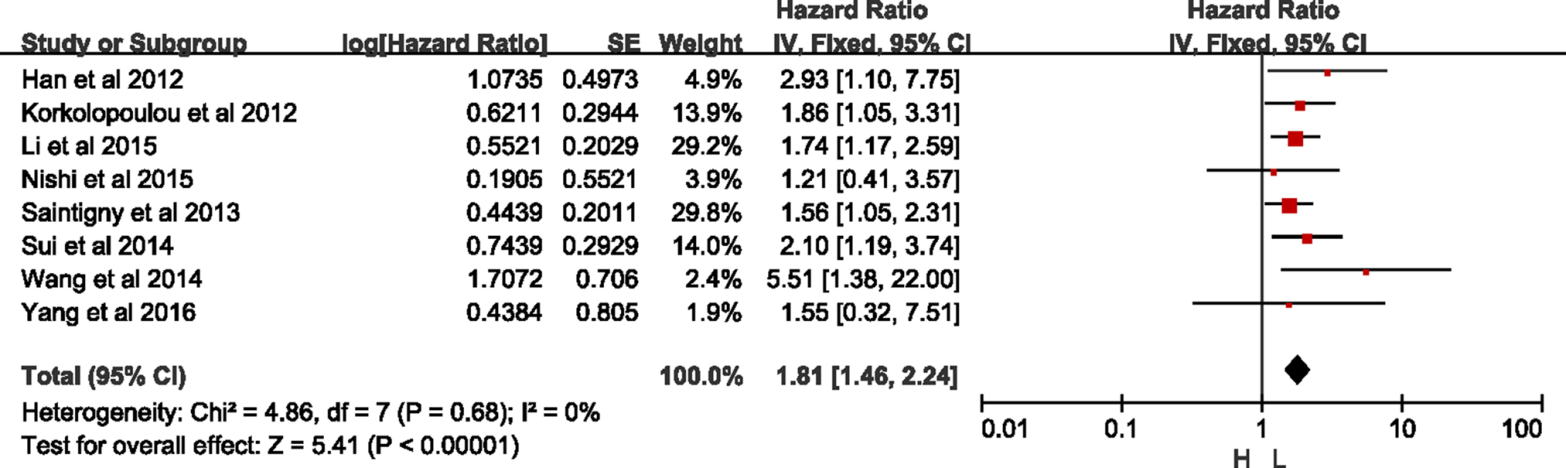
Meta-analysis of small sample size group

Both in big sample size study (sample sizes ≥300, HR = 1.76, 95% CI = 1.43–2.17, *p* < 0.00001; I^2^ = 0%, *p* = 0.63), and small sample size study (sample sizes < 300, HR = 1.61, 95% CI = 1.13–2.32, *p* = 0.009; I^2^ = 58%, *p* = 0.02) was consistently indicated that higher CXCR2 was correlated with shorten OS. But there was heterogeneity among the studies with small sample size (Table 2). Based on sensitivity presented in Supplementary Figure 5, study by Liang *et al.* [15] was excluded in analysis of small sample size group, the same as statistical analysis of Asian group. As what was shown in Figure 6, there was significant difference between high- and low-expression of CXCR2 without heterogeneity in small sample size group (HR = 1.81, 95% CI = 1.46–2.24, *p* < 0.01; I^2^ = 0%, *p* = 0.68).

## DISCUSSION

Cancer is still a large health problem worldwide. Although standard treatments have been utilized in most cancer patients, not all of patients derive benefit from these treatment strategies. It has been proven that low expression of CXCR2 was associated with increased tumor necrosis and the CXC chemokines receptors played a critical role in tumor either [19, 20].

Previous studies had demonstrated that chemokine interleukin-8 (CXCL8), the ligand of CXCR2, acted as autocrine and/or paracrine growth and proangiogenic factor for melanoma through CXCR2, inducing invasion and migration [21]. Study by Ha *et al.* reported that interaction between CXCL8 secreted by select cancer cells and CXCR2 in the tumor microenvironment is essential for cancer progression and metastasis by regulation cancer stem cell (CSC) proliferation and self-renewal [22]. Moreover, it was covered that CXCR2 engaged in Rho, Rac and mitogen-activated protein kinase signaling pathways which were connected to cell growth and migration [23, 24].

In our meta-analysis, it was presented that high expression of CXCR2 was significantly related to shorten OS and was a risk factor of OS. Among the studies included in quantitative synthesis, the synthetic analysis of 4 eligible studies reported that high CXCR2 expression was correlated with shorten recurrence-free survival (RFS). It was also shown that high CXCR2 expression was a risk factor of RFS. Likewise, subgroup analyses revealed that CXCR2 was an unfavorable predictor of OS in cancer. The result that high-level of CXCR2 was a risk factor of both OS and PFS, consistent with previous finding, strengthened the fact that low expression of CXCR2 predicted better efficacy of cancer therapy. Furthermore, we expanded the discussion on the comprehensive influence of several cancers rather than concrete category of cancer; moreover, subgroup analysis based on different type of cancer was also conducted. It was identified that CXCR2 was a risk factor for cancer both in synthetic analysis and subgroup analysis. Moreover, it was suggested that overexpression of CXCR2 in liver metastases from colon cancer was correlated to short disease-free and OS in the study conducted by Desurmont *et al.* [25]. Besides, study by Rezakhaniha *et al.* reported that CXCR2 expressions were correlated with shorter OS [26]. However, one important caveat that needs to be considered was that only one study was included in the meta-analysis of laryngeal squamous cell carcinoma, astrocytic tumors, pancreatic ductal carcinoma, clear-cell renal cell carcinoma and hepatocellular carcinoma, respectively, which might influence the reliability and veracity of some subgroup analyses. In addition, the unfavorable prediction of CXCR2 was performed solidly in different analysis model, ethnicity and sample size, which suggested that CXCR2 was a powerful prognostic predictor with a wide application in general population. Overall, according to the result of our meta-analysis, the better clinical management of cancer will be taken with the help of CXCR2 in the future. However, it was important to caveat that there was heterogeneity in subgroup analysis of Asian which indicated that the use of CXCR2 in Asian should be cautious. It was contradicted that study by Liang *et al.* [15] reported that overexpression of CXCR2 was a favorable prognosis predictor in Asian and study by Nishi *et al.* [19] and Yang *et al.* [28] reported that no significant difference was detected between high and low level of CXCR2 while the other studies conducted in Asian covered that overexpression of CXCR2 was a risk factor, which resulted the heterogeneity. Besides, the heterogeneity existed in small sample size studies might be located in the insufficient studies. Sensitivity analysis was conducted to detect the heterogeneity. In both subgroup analysis of groups with multivariate models, Asian group and small sample size with regard of OS, study by Liang *et al.* [15] was the source of heterogeneity, which reported that overexpression of CXCR2 was a favorable factor in esophageal adenocarcinoma and squamous cell carcinoma in terms of OS and was contradictory to previous studies.

The significant strength of our meta-analysis is that fully literature search was conducted and that no previous study illustrated the correlation between CXCR2 and cancers in the form of meta-analysis. Besides, the highlighted role of CXCR2 in several cancers was first explored synthetically in this meta-analysis. Moreover, several subgroup analysis were conducted to detect the influence of analysis models (univariate or multivariate models), sample size (< 300 or ≥ 300) and ethnicity (Asian and Caucasian) to the prognosis value of CXCR2. Besides, sensitivity was conducted to detect the heterogeneity exited in Asian group and small sample size group, and we found that the study by Liang *et al.* [15] was the source of heterogeneity. In this meta-analysis, it was first synthetically declared that CXCR2 was a potential and potent prognosis predictor in patients with cancer except for digestive tract cancer with wide application in clinic.

Nonetheless, our meta-analysis was not without limitations. For example, few studies were included to analyze RFS, which influenced the reliability of the results. Even if all 12 included studies have reported the OS, the number of relative study cohorts was still insufficient. Another 6 literatures [26–30] also presented the correlation between CXCR2 and OS, RFS, which all indicated that patients with lower CXCR2 expression had a better prognosis but without HR calculated in these studies. Moreover, the criteria to distinguish the expression level of CXCR2 were different in the enrolled studies, which might impair the veracity of the prognosis value of CXCR2. In addition, although there was unfavorable prognostic value of CXCR2 in digestive tract cancer in previous 4 studies [15–18] while the other one by Liang *et al.* [15] reported that CXCR2 indicates a better prognosis, in this meta-analysis, it was presented that no statistical significance was detected between high and low CXCR2 expression in subgroup analysis of digestive tract cancer in this meta-analysis. Hence, it was supposed that more clinical trials focused on the prognostic value of CXCR2 in digestive tract cancer should be conducted in the future. Last but not the least, patients in 7 studies included in this meta-analysis had no history of anticancer therapy while the others did not cover any concrete therapy. Although the conventional treatment of cancer might affect the expression of CXCR2, the analysis of conventional treatment were not conducted, which might affect the appliance of the results in this study.

In conclusion, CXCR2 was a risk factor of cancer prognosis with a wide application in terms of both OS and PFS except for digestive tract cancer, which could guide clinical treatment of various cancers. Further studies focused on more cancers especially for digestive tract cancer should be conducted.

## SUPPLEMENTARY MATERIALS FIGURES


