# Supplementary material for: The prognostic value of CXC chemokine receptor 2 (CXCR2) in cancers: a meta-analysis
Source: Oncotarget. 2017 Dec 11;9(19):15068–76. doi: 10.18632/oncotarget.23492 (PMC5871098; doi:10.18632/oncotarget.23492)
Supplement: Supplementary file 1 [file oncotarget-09-15068-s001.pdf]

The prognostic value of CXC chemokine receptor 2 (CXCR2) in cancers: a meta-analysis

SUPPLEMENTARY MATERIALS

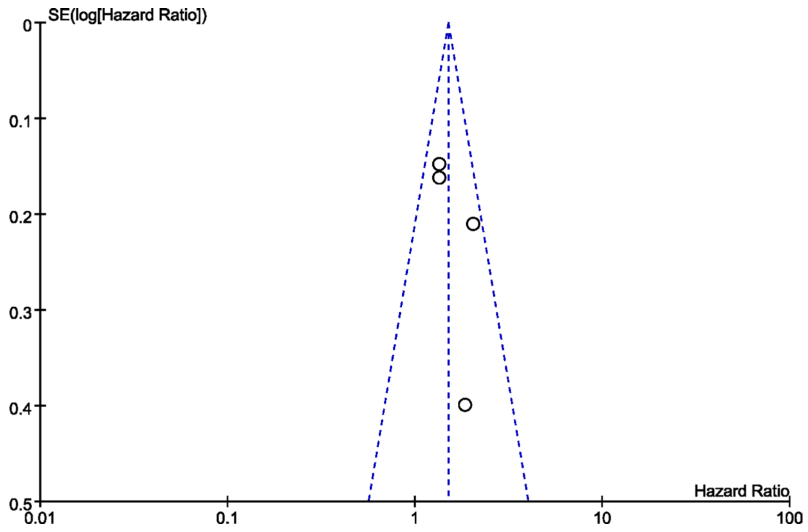

Supplementary Figure 1: Funnel plot of recurrence-free survival.

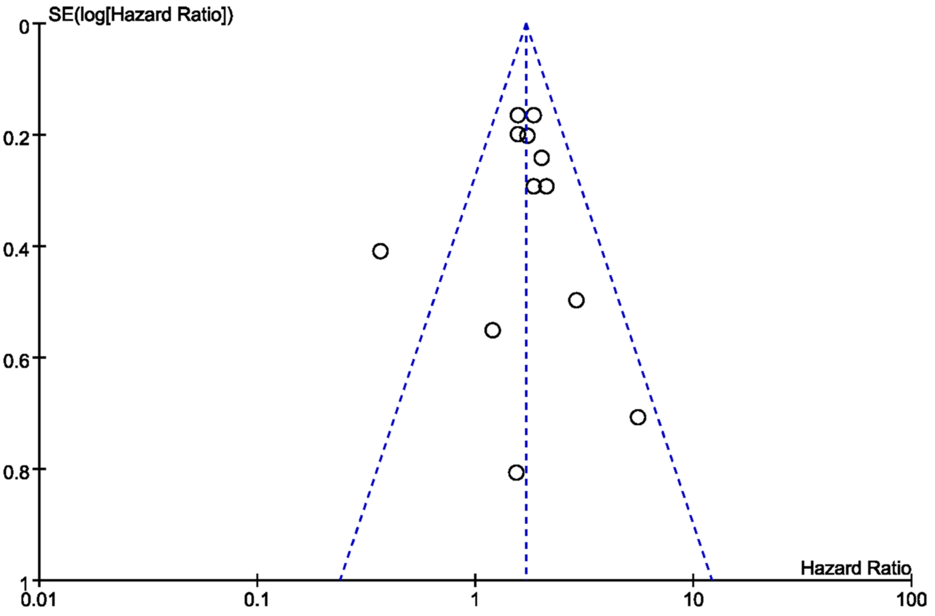

Supplementary Figure 2: Funnel plot of overall survival.

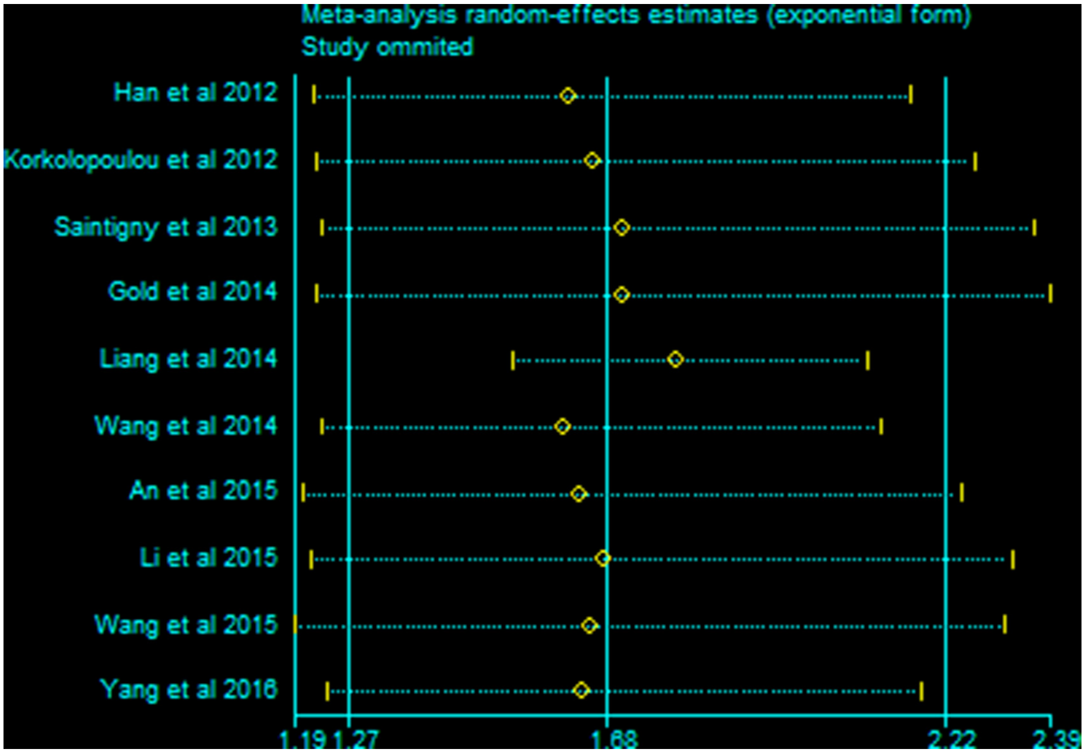

Supplementary Figure 3: Sensitivity analysis of subgroup analysis with regard of group with multivariate model.

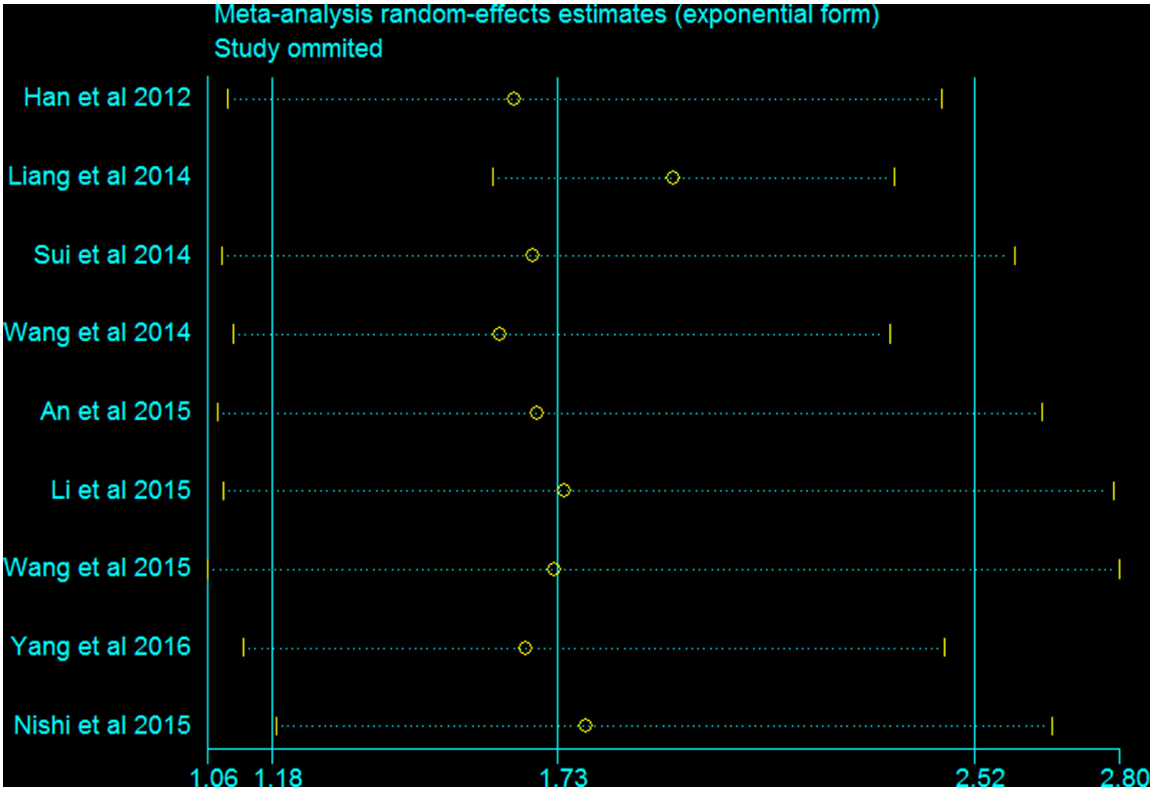

Supplementary Figure 4: Sensitivity analysis of subgroup analysis with regard of Asian group.

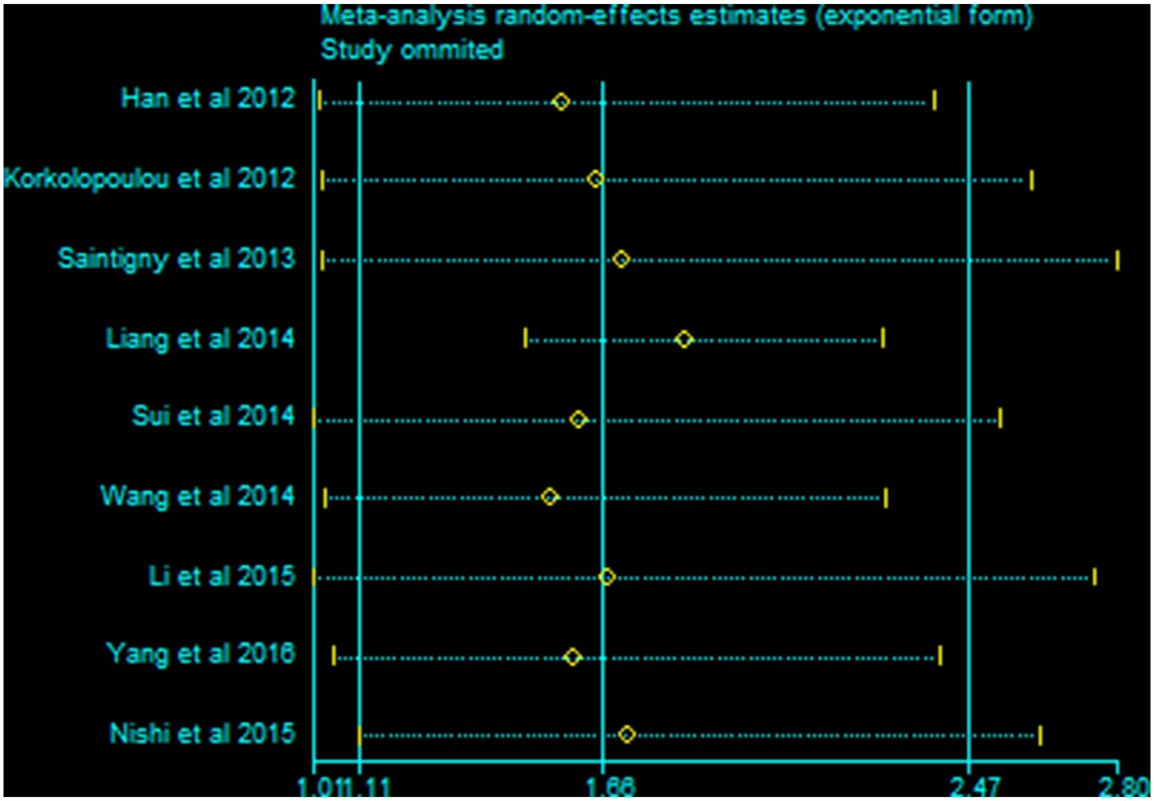

Supplementary Figure 5: Sensitivity analysis of subgroup analysis with regard of small sample size group.
